# P-840. Is *Staphylococcus lugdunensis* just another coagulase-negative *Staphylococcus*? A Comparative Study of community-acquired *Staphylococcus lugdunensis* and methicillin-susceptible *Staphylococcus aureus* Bacteremia

**DOI:** 10.1093/ofid/ofae631.1032

**Published:** 2025-01-29

**Authors:** Jessica Chung, Khin Wathan, Aye Honey Aung, Cherry Maung Maung Aye, Franklin Liu, Monica Ghitan, Edward Chapnick, Yu Shia Lin

**Affiliations:** Maimonides Health, Brooklyn, New York; Maimonides Health, Brooklyn, New York; Maimonides Health, Brooklyn, New York; Maimonides Health, Brooklyn, New York; Maimonides Health, Brooklyn, New York; Maimonides Medical Center, Brooklyn, New York; Maimonides Medical Center, Brooklyn, New York; Maimonides Medical Center, Brooklyn, New York

## Abstract

**Background:**

*Staphylococcus lugdunensis* (SL), a coagulase-negative staphylococcus (CoNS), usually a skin commensal, has demonstrated similar virulence to *Staphylococcus aureus*. Current studies have compared SL bacteremia (SLB) to *S. aureus* bacteremia, primarily hospital-onset methicillin-resistant *Staphylococcus aureus* bacteremia. Our study is the first to compare the clinical characteristics and outcomes between community-acquired SL bacteremia (SLB) and MSSA bacteremia (SAB).

**Table 1:**
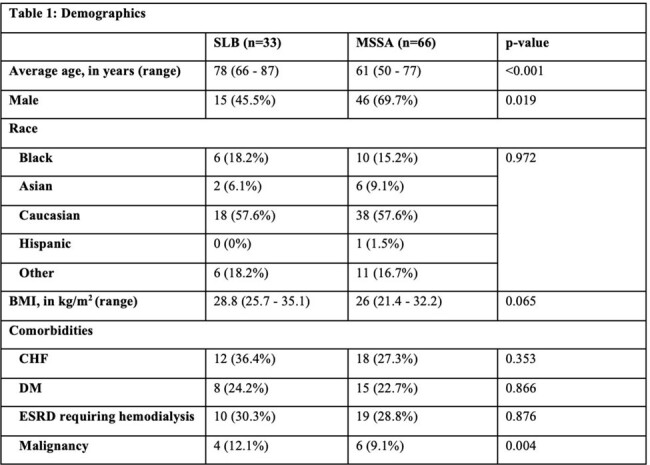
Patient Demographics

**Methods:**

This retrospective cohort study evaluated hospitalized adults with SLB and SAB at an urban tertiary care center from January 1, 2017 to December 31, 2022. CA-BSI was defined as at least one positive blood culture with SL or methicillin-susceptible *Staphylococcus aureus* (MSSA) within 72 hours of admission. Patients previously hospitalized within 30 days, on chemotherapy, or transferred from another facility were excluded. The primary outcome was in-hospital mortality rate with a secondary outcome of hospital length of stay (LOS). The Whitney Mann U test and chi-square test were used for the analysis.

Figure 2

**Figure:**
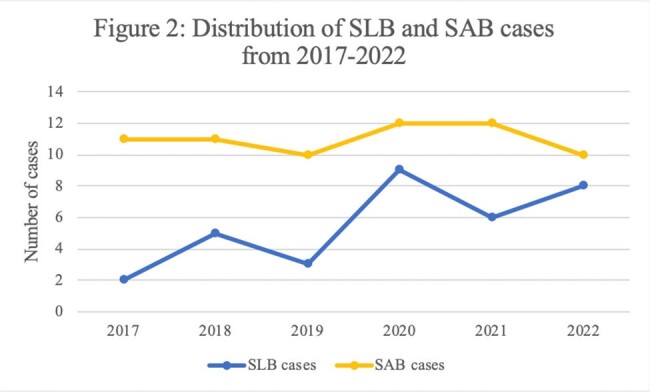

**Results:**

33 SLB patients and 66 SLB patients were included. The demographics of both groups are seen in Table 1. The SLB group was older and had a higher Charlson Comorbidity Index score compared to the SAB group. The incidence of SLB increased during the study period while the incidence of SAB remained the same (Figure 1). Two-thirds of the patients in both groups met sepsis criteria on presentation. 33% of SLB and 18% of SAB had a Pitt bacteremia score of ≥4 points. Endocarditis, skin and soft tissue infections, osteoarticular, line-related, and pneumonia are the primary sources of infections in SLB and SAB. There was no statistical difference in rates of ICU admission, need for ventilator support, need for renal replacement therapy, and length of stay between the two groups. In-hospital mortality was 15% for both groups.

**Conclusion:**

SL is a CoNS recognized as an emerging pathogen. The incidence of CA-SLB is increasing, particularly in older individuals with underlying comorbid conditions. The in-hospital mortality rate associated with SLB is as high as CA-SAB. Clinicians must recognize the high pathogenicity of SLB and implement comprehensive strategies to prevent morbidity and mortality.

**Disclosures:**

**All Authors**: No reported disclosures

